# Impact of vaccine supplies and delays on optimal control of the COVID-19 pandemic: mapping interventions for the Philippines

**DOI:** 10.1186/s40249-021-00886-5

**Published:** 2021-08-09

**Authors:** Carlo Delfin S. Estadilla, Joshua Uyheng, Elvira P. de Lara-Tuprio, Timothy Robin Teng, Jay Michael R. Macalalag, Maria Regina Justina E. Estuar

**Affiliations:** 1 Department of Mathematics, Ateneo de Manila University, Katipunan Ave., Brgy. Loyola Heights, 1102 Quezon City, Philippines; 2 Department of Psychology, Ateneo de Manila University, Quezon City, Philippines; 3 Department of Mathematics, Caraga State University, Butuan City, Philippines; 4 Department of Information Systems and Computer Science, Ateneo de Manila University, Quezon City, Philippines

**Keywords:** Optimal control, COVID-19 pandemic, Philippines, Non-pharmaceutical interventions, Vaccines

## Abstract

**Background:**

Around the world, controlling the COVID-19 pandemic requires national coordination of multiple intervention strategies. As vaccinations are globally introduced into the repertoire of available interventions, it is important to consider how changes in the local supply of vaccines, including delays in administration, may be addressed through existing policy levers. This study aims to identify the optimal level of interventions for COVID-19 from 2021 to 2022 in the Philippines, which as a developing country is particularly vulnerable to shifting assumptions around vaccine availability. Furthermore, we explore optimal strategies in scenarios featuring delays in vaccine administration, expansions of vaccine supply, and limited combinations of interventions.

**Methods:**

Embedding our work within the local policy landscape, we apply optimal control theory to the compartmental model of COVID-19 used by the Philippine government’s pandemic surveillance platform and introduce four controls: (a) precautionary measures like community quarantines, (b) detection of asymptomatic cases, (c) detection of symptomatic cases, and (d) vaccinations. The model is fitted to local data using an L-BFGS minimization procedure. Optimality conditions are identified using Pontryagin’s minimum principle and numerically solved using the forward–backward sweep method.

**Results:**

Simulation results indicate that early and effective implementation of both precautionary measures and symptomatic case detection is vital for averting the most infections at an efficient cost, resulting in $>99\%$ reduction of infections compared to the no-control scenario. Expanding vaccine administration capacity to 440,000 full immunizations daily will reduce the overall cost of optimal strategy by $25\%$, while allowing for a faster relaxation of more resource-intensive interventions. Furthermore, delays in vaccine administration require compensatory increases in the remaining policy levers to maintain a minimal number of infections. For example, delaying the vaccines by 180 days (6 months) will result in an $18\%$ increase in the cost of the optimal strategy.

**Conclusion:**

We conclude with practical insights regarding policy priorities particularly attuned to the Philippine context, but also applicable more broadly in similar resource-constrained settings. We emphasize three key takeaways of (a) sustaining efficient case detection, isolation, and treatment strategies; (b) expanding not only vaccine supply but also the capacity to administer them, and; (c) timeliness and consistency in adopting policy measures.

**Graphic Abstract:**

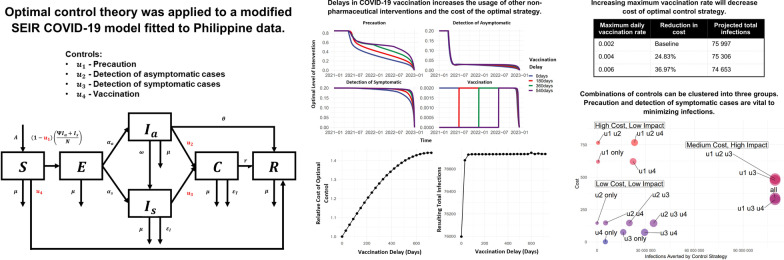

**Supplementary Information:**

The online version contains supplementary material available at 10.1186/s40249-021-00886-5.

## Background

In the year since the emergence of the global coronavirus disease 2019 (COVID-19) pandemic, national policies have had to decisively manage diverse issues of resource availability, institutional capacity, and collective behavioral change [1–3]. Striking the right balance of multiple strategies at the right time has been vital for implementing successful pandemic responses [4, 5]. Mathematical modelling has helped scientists and policymakers incorporate emergent discoveries about COVID-19 with existing knowledge to design effective interventions [6, 7].

In early 2021, the global introduction of vaccination as a viable counter to the disease prompts new analytical efforts. Regional inequalities introduce challenges to the global vaccine supply chain which may disrupt a straightforward vaccine rollout for a significant proportion of various national populations [8, 9]. Important questions emerge with respect to how governments may adequately adjust existing policies available for pandemic control in relation to multiple scenarios.

This paper undertakes an optimal control study of policies to control the COVID-19 outbreak in the Philippines, a developing country that may be particularly vulnerable to experiencing challenges to vaccine rollouts. Amidst large-scale preparations for the evaluation, selection, and distribution of vaccines, ongoing policies to respond to the pandemic continue to inform the Philippine government’s strategies for pandemic management [10]. Questions around their optimal implementation are particularly salient for developing countries that face heavier burdens from both the pandemic and overly restrictive quarantine measures [11]. This study therefore asks: How should the Philippine government implement existing strategies of community quarantine and case detection in conjunction with the introduction of vaccine rollouts?

## Related work

### Mathematical modelling for forecasting COVID-19 outbreaks

Since the beginning of the COVID-19 pandemic, the academic literature has witnessed a vast surge of modelling studies. Existing reviews highlight the importance of compartmental models of COVID-19, in connection with other models based on time series forecasting and machine learning [12, 13]. Compartmental models mathematically encode known and emerging information about the transmission dynamics of the disease and have been locally applied across numerous contexts around the world, including major sites of COVID-19 transmission like China, India, Brazil, the United States, and the United Kingdom [14–18].

Mathematical modelling efforts have been beneficial for forecasting and intervention assessment [19–21]. For instance, in the United Kingdom, a stochastic, age-structured transmission model was used to quantify the costs and mortalities of unmitigated outbreaks without interventions, highlighting the need for sustaining combined control efforts [22]. In another example, an age-structured model with social contact matrices was used to compare the impacts of different reopening strategies on the relative reduction of cases in different regions in China [23].

### Optimal control theory for modelling pandemic response

In this work, we utilize optimal control theory to model effective pandemic response. Optimal control theory refers to a field of study that deals with finding optimal solutions to a problem expressed in the form of a nonlinear dynamical system [24, 25]. This helps identify efficient methods of achieving desired outcomes, such as cost-effective infection control [15, 26].

Numerous studies have implemented optimal control theory toward similar end goals. In the absence of vaccines, most early studies focused on non-pharmaceutical interventions, including various combinations of rapid testing, contact tracing, and awareness campaigns [27–31]. Ullah and colleagues sought to disentangle the impacts of quarantine and case detection rates on exposed, critical, and hospitalized COVID-19 patients [32]. Other research modelled the effect of limited total testing resources, through the addition of an isoperimetric constraint to the optimal control problem [33].

Eventually, however, newer research was further able to consider the impacts of eventual vaccine availability. In an age-structured model, Bonnans and Gianatti studied minimization of the death toll, cost of confinement, and hospitalization peaks discussing a possible extension of their model when a vaccine becomes available [34]. Libotte and colleagues likewise explored programs for vaccine administration within a multi-objective setup, determining a set of Pareto optimal strategies that would minimize infections while also minimizing the number of vaccines needed [35].

### Responding to COVID-19 in the Philippines

The present work specifically investigates the dynamics of COVID-19 in the Philippines and aims to identify optimal strategies for efficiently controlling infections. We draw on existing modelling efforts by the national pandemic surveillance system [36] to derive realistic parameters which match existing epidemic trends and available intervention strategies in the country [37]. By deploying models informed by local parameters of the disease, we therefore aim to provide both theoretically optimal and contextually practical recommendations for policymakers [38].

In the Philippines, non-pharmaceutical interventions have primarily included phased community quarantines and mandated wearing of face masks [39]. Enhancements to the capacity of the health system to efficiently detect asymptomatic and symptomatic infectious individuals have also been key [40, 41]. In early 2021, imminent vaccine rollouts posed a salient new factor for pandemic control. We therefore sought to design optimal strategies for their distribution, and consider appropriate responses to potential obstacles which may arise in resource-constrained settings.

### Aims of the current study

Burgeoning scholarship points to rich global knowledge of the effectiveness and efficiency of various policy tools against the pandemic. However, both nationally specific impacts of the pandemic and the limitations faced by intervening bodies highlight the importance of grounding optimal control analysis in the local context.

In this view, the present work therefore aims to achieve the following goals. First, we frame pandemic interventions with vaccinations as an optimal control problem to identify scenarios for effective pandemic control. Second, we explore various vaccination scenarios featuring both delays and expansions of vaccine administration. This enables a future-oriented analysis of how local policymakers may compensate for unforeseen developments in the global supply chain. Finally, we perform a systematic ablation analysis, whereby we restrict various combinations of available controls to model more limited control scenarios.

## Methods

### Model formulation

**Fig. 1:**
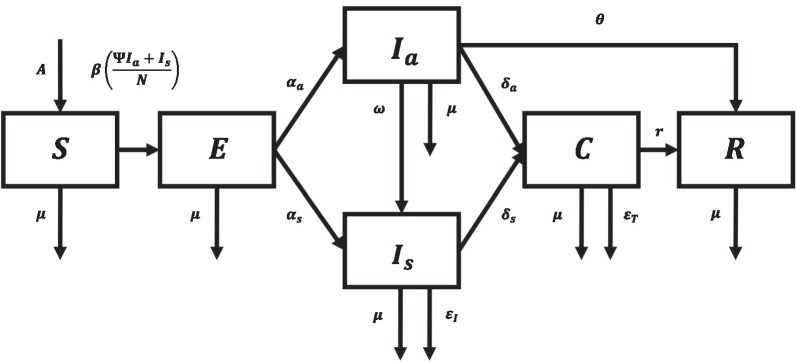
Diagram of the compartmental model. Disease progression from the susceptible (*S*) compartment through the exposed (*E*), asymptomatic $(I_a)$ and symptomatic $(I_s)$ infectious, confirmed (*C*), and removed (*R*) compartments

To capture the local dynamics of COVID-19 transmission in the country, we form a model that utilizes the local incidence data from the Department of Health-Epidemiology Bureau (DOH-EB) [42]. The COVID-19 model utilizes six compartments to subdivide the population: susceptible (*S*), exposed (*E*), infectious but asymptomatic $(I_a)$, infectious and symptomatic $(I_s)$, confirmed (*C*), and removed (*R*). These compartments are governed by the epidemic flow as illustrated in Fig. 1. Compartment *S* consists of individuals who have not been infected with COVID-19 but may contract the disease once exposed to the virus. Compartment *E* consists of individuals who have been infected but are still within the latency stage of the disease. These individuals will eventually become infectious, and categorized into two compartments depending on the presence ($I_s$) or absence ($I_a$) of symptoms. Once detected, these infectious individuals will move to compartment *C*, where they will be included among the *active cases*. Individuals in this compartment are assumed to be isolated, and hence not capable of infecting the susceptible population, and receiving treatment while in isolation. Lastly, compartment *R* consists of individuals who have acquired immunity from the disease. We assume that those who have recovered from the disease acquire permanent immunity and therefore move to compartment *R*.

The movements of individuals toward and out of the different compartments are governed by several parameters of the model. The transmission rate $\beta$ is a function of the disease transmission rate $\beta _0$, based on an assumed basic reproduction number $R_0$, and a reduction factor $(1 - \lambda )$. The parameter $\lambda$ reflects the effect of community quarantine imposed by the government, as well as the degree of compliance to minimum health standards, which includes practicing proper hygiene, social distancing, and wearing protective face coverings. Moreover, the parameter $\psi$ accounts for the infectiousness of asymptomatic individuals relative to those who have symptoms. The rates of transfer to the two infectious compartments, $\alpha _a$ for asymptomatic and $\alpha _s$ for symptomatic, are both dependent on the incubation period $\tau$ of the virus.

Other parameters in the model include the constant recruitment rate *A* into the *S* compartment, which is driven by new births in the population. To account for deaths by natural causes, a constant rate of $\mu$ per unit time is applied to all compartments in the model. In addition, deaths due to the disease are included through the parameters $\epsilon _I$ and $\epsilon _T$, affecting the infectious symptomatic and confirmed compartments, respectively.

By taking into account the above assumptions, a mathematical model is developed, which can be described by the following system of six ordinary differential equations:

1 $$\begin{aligned} {\left\{ \begin{array}{ll} \dfrac{dS}{dt} = A - \beta S \dfrac{\psi I_a + I_s}{N} - \mu S, S(0)\ge 0, \\ \dfrac{dE}{dt} = \beta S \dfrac{\psi I_a + I_s}{N} - (\alpha _a + \alpha _s + \mu )E, E(0)\ge 0, \\ \dfrac{dI_a}{dt} = \alpha _a E - (\mu +\omega +\delta _a+\theta ) I_a, I_a(0)\ge 0, \\ \dfrac{dI_s}{dt} = \alpha _s E+\omega I_a-(\mu +\epsilon _I+\delta _s)I_s, I_s(0)\ge 0, \\ \dfrac{dC}{dt} = \delta _a I_a+ \delta _s I_s - (\mu +\epsilon _T+r) C, C(0)\ge 0, \\ \dfrac{dR}{dt} = \theta I_a + r C - \mu R, R(0)\ge 0, \\ \end{array}\right. } \end{aligned}$$

where $\beta = \beta _0 (1-\lambda )$, $\alpha _a = \frac{c}{\tau }$, $\alpha _s = \frac{1-c}{\tau }$, $N = S + E + I_a + I_s + C + R$. The functions *S*, *E*, $I_a$, $I_s$, *C*, and *R* are differentiable real-valued functions on ${\mathbb {R}}$. Moreover, all parameters are nonnegative constants.

### Parameter values

The values of various parameters were determined using several sources and methods. Local COVID-19 data [42] were used in calculating detection rate ($\delta _a,\delta _s$), post-detection recovery rate (*r*), and death rate of COVID-19 cases ($\epsilon _I$, $\epsilon _T$). The recruitment rate (*A*) and natural death rate ($\mu$) are calculated from population data [43–45]. For the other parameters which cannot be computed directly from data, we rely on references that estimate their values. The basic reproduction number of COVID-19 ($R_0$) and relative infectiousness of asymptomatic cases ($\psi$) are based on estimates by the US Centers for Disease Control [46]. The incubation rate ($\tau$) and symptomatic transition ($\omega$) are obtained from reports by the World Health Organization [47, 48]. The proportion of asymptomatic cases (*c*) is from Mizumoto et al. [49] that studied the COVID-19 outbreak at the Diamond Princess cruise ship.

**Fig. 2:**
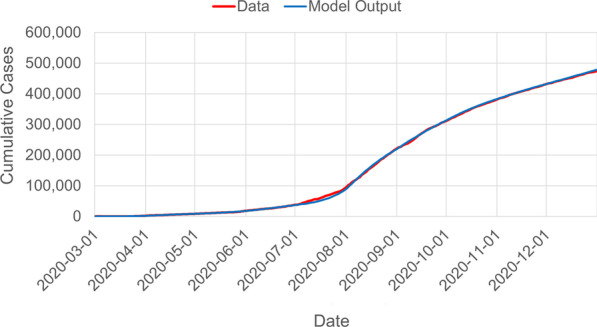
Fit of model output to data following L-BFGS optimization procedure

We fit the model output to data by employing a curve-fitting algorithm to estimate the value of the transmission reduction rate ($\lambda$), the initial values for the exposed (*E*(0)), infectious asymptomatic ($I_a(0)$), and infectious symptomatic ($I_s(0)$). In particular, the constrained L-BFGS optimization procedure [50] was utilized to minimize the sum of squared errors between the model output and the empirical time series. The parameter $\lambda$ is fitted on a per-month basis starting from March 2020, to coincide with the changes in the disease dynamics and the corresponding transmission reduction policies implemented by the government that tends to be updated monthly [51]. The output is a vector of best-fit transmission reduction parameters $[\lambda _1,\lambda _2,...,\lambda _n]$ where *n* is the number of months since March 2020. This fitting procedure is utilized to produce forecasts for the Philippine COVID-19 epidemic [36]. Following the above parametrization, our model fits well to the Philippine data for cumulative cases of COVID-19 (Fig. 2). Table 1 summarizes the parameter values used in the model.

**Table 1:** Summary of parameter values for the COVID-19 model

Variable	Description	Value	Unit	Source
$R_0$	Basic reproduction number	4.0000	None	[46]
*A*	Recruitment rate	1309.1	1/day	[43, 45]
$\mu$	Natural death rate	$4.0548 \times 10^{-5}$	1/day	[44]
$\beta _0$	Baseline transmission rate	0.4343	1/day	[46]
$\lambda$	Transmission reduction	0.6500 to 0.8500	1/day	[36]
$\psi$	Relative infectiousness of asymptomatic cases	1.0000	None	[46]
$\tau$	Incubation period	5.0000	Day	[48]
*c*	Proportion of asymptomatic infections	0.1800	None	[49]
$\omega$	Symptomatic transition	0.3300	1/day	[47, 49]
$\theta$	Recovery rate of asymptomatic	0.0714	1/day	[42]
$\delta _a$	Detection rate for asymptomatic	0.1000 to 0.2000	1/day	[42]
$\delta _s$	Detection rate for symptomatic	0.1000 to 0.2000	1/day	[42]
*r*	Post-detection recovery rate	0.0855	1/day	[42, 48]
$\epsilon _I$	COVID-19 death rate, undetected	0.0018	1/day	[42]
$\epsilon _T$	COVID-19 death rate, detected	0.0018	1/day	[42]

### Model with optimal control

We explore four control strategies to mitigate the COVID-19 epidemic-precautionary measures, detection of asymptomatic cases, detection of symptomatic cases, and vaccination. The definitions of each of these controls and how they are incorporated in the model are given below:

1. Precautionary measures ($u_1(t)$) refer to government-led efforts to inhibit possible contacts between susceptible and infectious individuals by regulating public gatherings, closing schools, suspending office work, enforcing adherence to health protocols such as social distancing, mask-wearing, hand-washing, etc. This control affects the transmission rate $\beta$ and is incorporated in the model as a factor ($1-u_1(t)$), replacing ($1 - \lambda$). The value of $u_1(t)$ represents the effort of precaution at time *t*. A value of 0 indicates that no precautionary measure is being practiced, and a value of 1 indicates full effort on precaution prohibiting any form of infection.
2. Detection of asymptomatic cases ($u_2(t)$) entails identifying and isolating infectious individuals who do not have symptoms of COVID-19. This may be done through laboratory tests such as reverse transcription-polymerase chain reaction (RT-PCR) to determine whether an individual is infectious or not. A positive case is taken to be immediately followed by isolation at home or in a dedicated quarantine facility to prevent transmission. It is assumed, therefore, that after an individual is confirmed to have COVID-19, s/he is not able to infect susceptible individuals. We incorporate this control to the model by replacing $\delta _a$ with a time-varying control function $u_2(t)$. The value of $u_2(t)$ represents the effort of testing and isolation at a given time *t*. A value of 0 indicates the absence of testing and isolating, and a value of 1 indicates testing and isolating all infectious asymptomatic individuals on a given unit of time.
3. Detection of symptomatic cases ($u_3(t)$) follows the same definition as the detection of asymptomatic cases but applied to individuals that exhibit symptoms of COVID-19. We replace $\delta _s$ by $u_3(t)$ to incorporate this control to the model. Similarly, a value of 0 of this control indicates the absence of effort to test and isolate symptomatic individuals while a value of 1 indicates full testing and detection of all symptomatic individuals on a given unit of time.
4. Vaccination ($u_4(t)$) refers to the full inoculation of susceptible individuals for them to acquire protection against COVID-19 infection or protection against a severe case of the disease. We assume in this paper that vaccines give protection against infection, that is, an individual who is fully vaccinated gets immunity to COVID-19 over the period considered. Multiple vaccines with varying effectiveness rates have been identified for use against COVID-19 such as those developed and manufactured by Pfizer-BioNTech, Moderna, Sinovac, etc. We consider the average effectiveness rate of the vaccines weighted by the usage, denoted by $\sigma$, where $0\le \sigma \le 1$. To incorporate vaccination in the model, we add a rate of transfer from compartment *S* to *R* equal to $\sigma u_4(t)$. The value of $u_4(t)$ represents the effort of vaccination for the susceptible population. A value of 0 represents no vaccination efforts while a value of 1 represents vaccination of all susceptible individuals on a single unit of time.

Our goal is to identify the optimal strategy for limiting the spread of SARS-CoV-2 in a population using minimal cost of controls. In this study, the optimal control problem minimizes the number of asymptomatic ($I_a$) and symptomatic individuals ($I_s$) and the control costs. The controls are expressed in quadratic forms to incorporate nonlinear costs for the implementation of each control and to ensure the convexity of the cost function. This is a common form of an objective functional in optimal control problems [25, 52]. The objective functional is represented by:

2 $$\begin{aligned} J(\vec {u}) = \int _{t_0}^{t_f} \bigg (I_a(t) + I_s(t) + w_1 u_1^2(t) + w_2 u_2^2(t) + w_3 u_3^2(t) + w_4 u_4^2(t)\bigg ) \,dt, \end{aligned}$$

where $t_0$ and $t_f$ represent January 1, 2021 and December 31, 2022 respectively, reflecting a 2-year period. The parameters $w_i, i=1,2,3,4,$ account for the relative costs of implementing controls $u_i$. They represent the weights of corresponding terms in the integrand and their importance in the optimal control problem.

We aim to identify $u_i^*(t), i=1,2,3,4,$ such that

3 $$\begin{aligned} J\left( u_1^*, u_2^*, u_3^*,u_4^*\right) = \min _{{\mathscr {U}}}J\left( u_1, u_2, u_3,u_4\right) , \end{aligned}$$

where for Lebesgue integrable $u_i$,

$$\begin{aligned} {\mathscr {U}} = \left\{ \left( u_1, u_2, u_3,u_4\right) | u_{i}^{\mathrm{{min}}} \le u_i(t)\le u_{i}^{\mathrm{{max}}}, t_0\le t \le t_f \right\} . \end{aligned}$$

Here, $u_{i}^{\mathrm{{min}}}$ and $u_{i}^{\mathrm{{max}}}$ are the lower and upper bounds of the control $u_i$, representing minimum and maximum implementation efforts.

The constraints of the optimal control problem are given by:

4 $$\begin{aligned} {\left\{ \begin{array}{ll} \dfrac{dS}{dt} = A - (\beta _0(1- u_1)) S \dfrac{\psi I_a + I_s}{N} - \sigma u_4 S - \mu S, S(0)\ge 0, \\ \dfrac{dE}{dt} = \beta _0(1- u_1) S \dfrac{\psi I_a + I_s}{N} - (\alpha _a + \alpha _s + \mu )E, E(0)\ge 0, \\ \dfrac{dI_a}{dt} = \alpha _a E - (\mu +\omega +u_2+\theta ) I_a, I_a(0)\ge 0, \\ \dfrac{dI_s}{dt} = \alpha _s E+\omega I_a-(\mu +\epsilon _I+u_3)I_s, I_s(0)\ge 0, \\ \dfrac{dC}{dt} = u_2 I_a+ u_3 I_s - (\mu +\epsilon _T+r) C, C(0)\ge 0, \\ \dfrac{dR}{dt} = \theta I_a + r C + \sigma u_4 S - \mu R, R(0)\ge 0, \\ \end{array}\right. } \end{aligned}$$

where

$$\begin{aligned} N = S + E + I_a + I_s + C + R. \end{aligned}$$

The existence of the optimal solution can be shown using standard results in optimal control theory [25, 52]. The necessary convexity of the integrand of the objective functional, positive definiteness of system (4), and the linear dependence of the state differential equations to the controls are satisfied in our model.

We apply Pontryagin’s minimum principle [24] to determine the necessary conditions using the optimality system for our problem (see Additional file 1: Appendix). This system is a two-point boundary problem with initial conditions for the state variables and terminal conditions for the adjoint variables. The solutions are solved numerically using a Runge–Kutta fourth-order scheme. The state variables are solved forward in time while the adjoint variables are solved backwards, referred to as Forward–Backward Sweep Method [25]. We update the controls using a convex combination of the latest and previous values. This process is iterated until the updates in the control values are very small or less than the machine epsilon.

The initial state values are computed using a combination of data and model fitting. We relied on model fitting to data to get the values for $E(0),I_a(0)$ and $I_s(0)$ [36]. The initial value for confirmed cases (*C*(0)) is based on data from the Department of Health [42]. The initial number of removed individuals (*R*(0)) is assumed to be higher than the detected recoveries on January 1, 2021, to include recoveries from undetected asymptomatic cases. We estimate that this is equal to 450,000, consistent with the output of our model [36]. Lastly, the initial susceptible population is estimated to be equal to the whole population minus the assumed values for the other compartments. Table 3 lists the initial state values used in our simulations.

**Table 2:** Lower ($u_{i}^{\mathrm{{min}}}$) and upper ($u_{i}^{\mathrm{{max}}}$) bounds for control strategies representing available effort in the Philippines

Control	$u_{i}^{\mathrm{{min}}}$	$u_{i}^{\mathrm{{max}}}$	Sources
Precaution ($u_1$)	0	0.850	[36]
Detection of asymptomatic ($u_2$)	0	0.200	[42]
Detection of symptomatic ($u_3$)	0	0.200	[42]
Vaccination ($u_4$)	0	0.002	[10]

**Table 3:** Initial values for model states

State	Initial value	Sources
Susceptible (*S*)	108,960,983	[45]
Exposed (*E*)	7000	[36]
Infectious asymptomatic ($I_a$)	500	[36]
Infectious symptomatic ($I_s$)	9000	[36]
Confirmed cases (*C*)	57,833	[42]
Removed (*R*)	450,000	[36, 42]

We fix $u_{i}^{\mathrm{min}}=0$ for $i\in \{1,2,3,4\}$ while upper bounds of the controls are varied to reflect the realistic maximum efforts that can be achieved with each control. Results from model fitting to data (see Table 1) show that the highest value for precaution is 0.85. Direct computations from epidemiological data provided by the Department of Health [42] show that the minimum monthly average duration of detection, from symptom onset to confirmation of test results, may take 5 days. We take the inverse of this duration as our upper bound for both detection controls, hence $u_{2}^{\mathrm{{max}}} = u_{3}^{\mathrm{{max}}} = 0.2$. Lastly, the upper bound for vaccination ($u_4$) is based on government proclamations [10]. The boundaries for the control values are summarized in Table 2.

The weight parameters $w_i, i=1,2,3,4,$ are adjusted to balance the terms in the integrand of the cost function (2). These parameters reflect the total costs and payoffs of implementing each control strategy including the cost of the products used (test kits, vaccines, masks, etc.), operational costs (personnel salary, rent, procurement, refrigeration units, etc.), opportunity costs for the economy due to lockdowns, and so forth. To determine the values of the weight parameters, we consider the fact that the upper bounds for the controls already reflect the realistic and achievable efforts that the Philippine government can exert, given the historical and prospective cost and availability of each control. Recall that the upper bounds for precaution, detection of asymptomatic cases, and detection of symptomatic cases are based on data, and the upper bound for the vaccination control is based on government targets. Based on this, we assume that a lower $u_i^{max}$ signifies a relatively higher implementation cost for the *i*-th control as implementing the control beyond this upper bound is not readily available to the government. Given this, we rescale the terms in the cost function (2) by equating the weight parameters to the inverse of the maximum allowable effort for each control ($w_1=1/0.85, w_2=w_3=1/0.2, w_4 = 1/0.002$). This improves the balance of the terms in the cost function and reduces the bias to implement controls that have lower upper bounds.

Limited information is available as of writing to estimate the vaccine effectiveness parameter ($\sigma$) for COVID-19. Moreover, various vaccines with different effectiveness rates will be deployed in the Philippines as they become available [10], making it more difficult to give a realistic estimate of this parameter. The best alternative is to equate this to the pooled effectiveness of vaccines for a similar disease such as influenza, which is at $\sigma =0.7$ [53].

## Results

### Optimal control strategies for COVID-19 in the Philippines

**Fig. 3:**
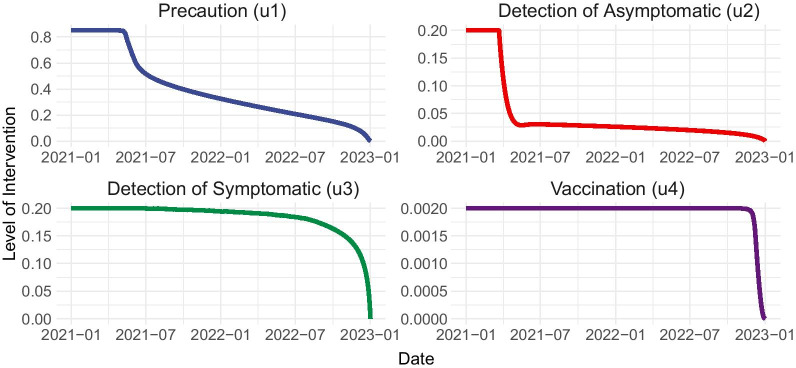
Optimal control strategy for the COVID-19 epidemic in the Philippines

Solving the stated optimal control problem in Eqs. 2–4 generates the optimal levels of precaution ($u_1$), asymptomatic detection ($u_2$), symptomatic detection ($u_3$), and vaccination ($u_4$) over the 2-year period from January 1, 2021 to December 31, 2022 (Fig. 3). Notably, the maximum feasible vaccination rate must be sustained throughout the entire 2-year period. Symptomatic detection must likewise maintain a high value close to the maximum feasible value, lowering slightly in the first 6 months of 2021. Asymptomatic detection and precaution must likewise be implemented at their maximum respective values early in 2021. But asymptomatic detection may be eased to nearly zero by the second quarter of 2021, while precaution eases a bit by the second half of 2021, then is gradually reduced throughout the remainder of the 2 years under consideration.

**Fig. 4:**
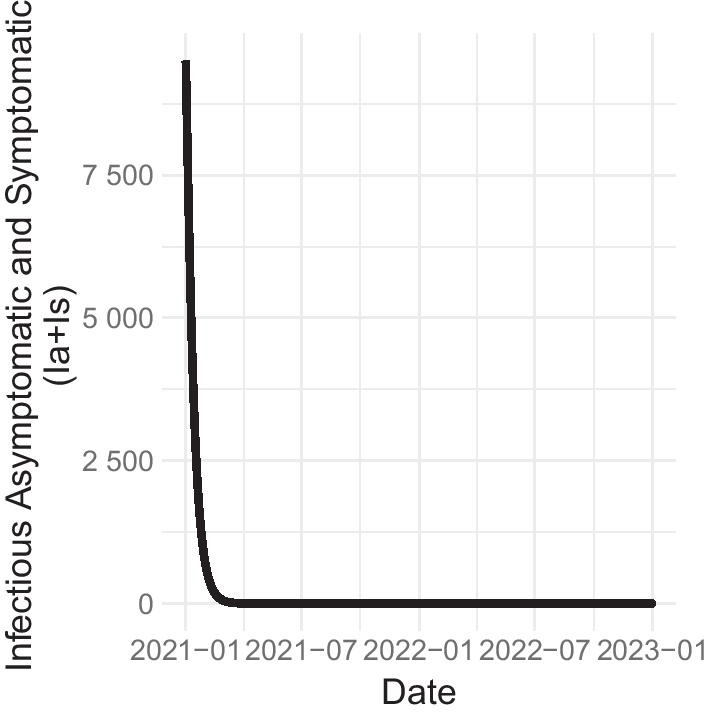
Total infectious individuals ($I_a + I_s$) with full implementation of the optimal control strategy

We also simulate a no-control scenario by setting the controls to 0 throughout the 2-year period. A dramatic difference is observed between the with-control and without-control conditions (Fig. 4). Without controls, a peak number of 100 million infectious individuals is achieved within the first quarter of 2021. Meanwhile, with the optimal implementation of all controls, the total number of running infections is driven down quickly in early 2021, without ever breaching the 10,000-mark. After the full-throttle implementation of all controls in early 2021, sustained efforts at detecting symptomatic individuals and proactively vaccinating susceptible populations may thus be sufficient to prevent the infected population from rising. So long as the latter strategies are maintained, the majority of the population may slowly ease stringent distancing rules and fewer resources need to be urgently allocated to asymptomatic detection.

### Policy impacts of vaccine delays

**Fig. 5:**
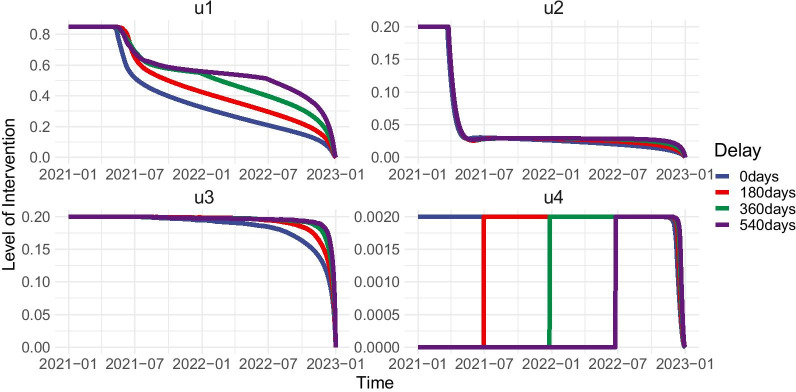
Delay of vaccine availability increases effort for other controls in the optimal strategy. $u_1$-Precautionary measures, $u_2$-detection of asymptomatic cases, $u_3$-detection of symptomatic cases, $u_4$-vaccination

With the bottleneck in the global supply of vaccines, it is of chief concern when a country can start vaccinating its population. It is not far-fetched for countries to experience delays in vaccination which in turn, would have an effect on policy. Here, we look into the impact of vaccine delay on the optimal control strategy. To achieve this, we add the following constraint to the optimal control problem 2–4:

$$\begin{aligned} u_4(t)=0, \quad t\in [t_0,t_d], \quad t_0\le t_d \le t_f, \end{aligned}$$

where $t_d$ is the vaccine delay in days. We first solve for the optimal control profiles given vaccine delays of 180, 360, and 540 days (Fig. 5). Results reveal that increased efforts on the other controls become necessary given longer delays in vaccination. Primarily, precautionary measures should compensate when vaccines are delayed. Detection of symptomatic infectious individuals should also be strengthened for mitigation if vaccine rollout is slowed down.

To further evaluate the effect of vaccine delay, we compute the cost of the optimal strategy in each scenario. The cost of the control strategy (${\mathscr {C}}$) is defined as the integral of the last four terms in the cost function of the optimal control problem over the time period, specifically:

5 $$\begin{aligned} {\mathscr {C}} = \int _{t_0}^{t_f} \bigg (w_1 u_1^2(t) + w_2 u_2^2(t) + w_3 u_3^2(t) + w_4 u_4^2(t)\bigg ) dt. \end{aligned}$$

**Fig. 6:**
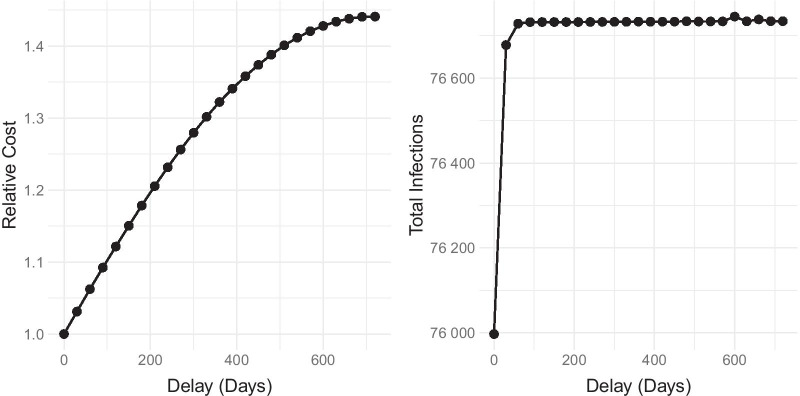
Effects of delays in initiation of vaccination strategies on the relative cost of overall strategy (left) and resulting total infections over the 2-year period (right)

We observe that the optimal strategy in the no-delay scenario has the least cost and will also result in the least number of total infections. Given this, we decided to compare the cost of the optimal strategy in the scenarios with vaccine delay relative to the no-delay scenario. Specifically, given vaccine delays of 30*k* days, where $k\in \{0,1,2,3,...,24\}$, we examine the resulting relative cost and total infections of the optimal control strategy (Fig. 6).

Based on our simulations, longer delays in vaccination result in higher relative costs of the optimal strategy. For example, delaying the vaccines by 180 days will result in an $18\%$ increase in cost, and delaying the vaccine by 360 days will increase the cost by $32\%$, due to the compensation of the other controls. We also observe that delaying the vaccine by 60 days or more will only increase the total infections in the optimal strategy by a relatively small amount ($<1000$ additional infections). These two findings suggest that while the number of infections can still be effectively managed when vaccines are delayed, vaccine delay may pose more deleterious effects on the economy than on the overall health status of the population, even if the optimal strategy is implemented and new cases are minimized.

### Policy impacts of expanding vaccine supply

Recall that the upper bound for the vaccination control ($u_{4}^{\mathrm{{max}}}$) was fixed based on the vaccination plan by the local government. Note however that the actual vaccine capacity is unknown and is dependent on negotiations and supply. Here, we want to look into whether increasing vaccine supply will have a significant effect on the optimal control strategy. To discern this relationship, we modify the value of $u_{4}^{\mathrm{{max}}}$ to double and triple the initial value. The value of the weight parameter $w_4$ is equal to $1/u_{4}^{\mathrm{{max}}}$ in each scenario.

**Fig. 7:**
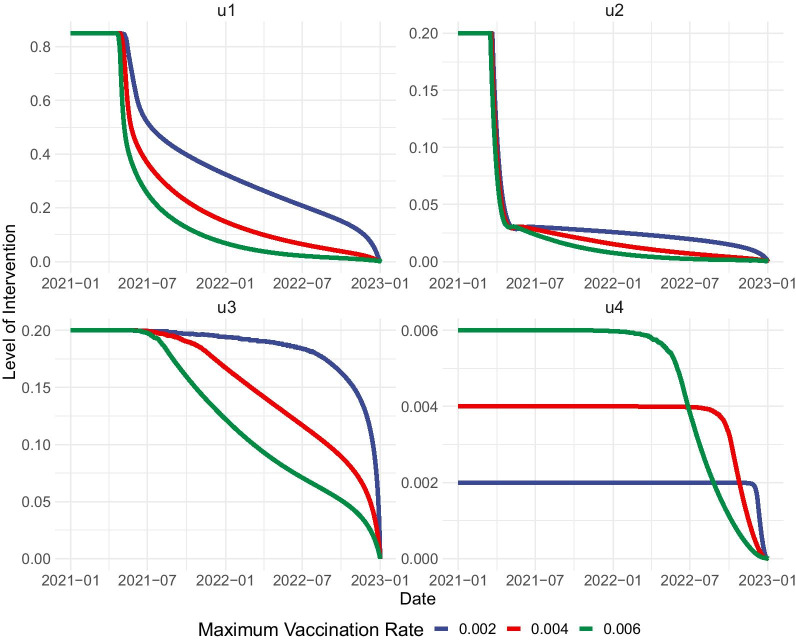
Increasing the upper bound for vaccination control may allow for shorter and lighter precautionary measures and testing. $u_1$-Precautionary measures, $u_2$-detection of asymptomatic cases, $u_3$-detection of symptomatic cases, $u_4$-vaccination

**Fig. 8:**
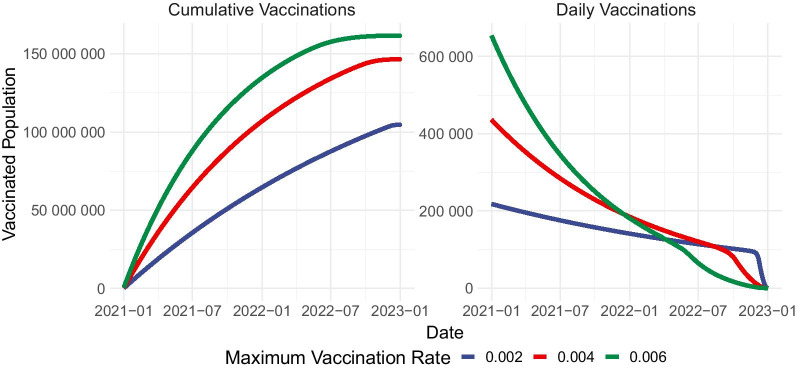
The optimal number of complete vaccinations (daily and cumulative) for different values of upper bound for vaccination

We solve for the optimal control profiles and the resulting number of vaccinations if $u_{4}^{\mathrm{{max}}}=0.002,0.004$ or 0.006 (Figs. 7, 8). We observe that increasing the vaccine capacity will have a significant impact on the optimal control strategy. For the three scenarios considered, the maximum vaccination effort must be utilized for almost the entire period, but vaccination effort is eased earlier if the vaccine capacity is larger. Another important consequence of increasing vaccine capacity is the earlier relaxation of the other controls, mainly precautionary measures and detection of symptomatic cases.

Comparing the relative cost and the resulting total infections reveals that increasing the vaccine capacity by double or triple the initial amount will reduce the cost of the optimal strategy (Table 4). We observe a 25% cost reduction when vaccine supply is doubled, and 37% cost reduction when vaccine supply is tripled, coupled with a slight reduction in the total number of infections. This reinforces the proposition that dedicating more resources to vaccinations is more favorable in the long run owing to the reduced efforts necessary for implementing the other interventions.

**Table 4:** Total infections and relative cost for optimal control strategy when upper bound for vaccination ($u_{4}^{\mathrm{{max}}}$) is increased

$u_{4}^{\mathrm{{max}}}$	Relative cost	Total infections
0.002	1.0000	75,997
0.004	0.7517	75,306
0.006	0.6303	74,653

### Managing cost and impacts of pandemic control strategies

Finally, to integratively consider the dynamics of all interventions, we analyzed the results of a control setup featuring all interventions in conjunction with ablated control scenarios featuring various subsets of the proposed controls. We compared outcomes for optimized single control, dual control, and triple control strategies to simulate scenarios when the other controls are not available, as well as to shed light on their contributions to controlling the epidemic, and highlight the significance of implementing all four in concert. To do this, controls that are not being implemented in each scenario are fixed at 0. Full details on various control profiles are available in Additional file 1: Appendix.

**Fig. 9:**
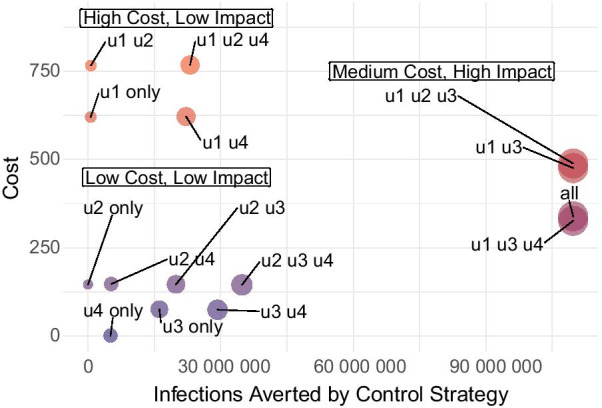
A menu of control strategies visualized according to infections averted (*x*-coordinate) and cost (*y*-coordinate). Points are sized by infections averted, such that larger points symbolize control strategies that avert more infections. Points are also colored by cost, such that bluer points incur lower costs, and redder points incur higher costs. $u_1$-Precautionary measures, $u_2$-detection of asymptomatic cases, $u_3$-detection of symptomatic cases, $u_4$-vaccination

We compare all possible combinations of the controls in terms of both their cost and the infections averted relative to the no-control scenario (Fig. 9). Intuitively, an ideal scenario entails low cost and high infections averted. Strikingly, the control scenarios appear to cluster into three major categories. First are *low cost, low impact* strategies, which do not entail high costs, but also do not effectively curb infections. These correspond to intervention programs that do not mobilize sufficient resources to address the health crisis and subsequently do not achieve the desired impact. We observe here that this cluster of intervention combinations primarily exclude precautionary measures like community quarantines, meaning various scenarios implementing only vaccinations and case detection strategies. This entails that, even if these strategies might be less costly-especially from an economic perspective-than prolonged lockdowns, they may not be sufficient on their own to control outbreak trajectories. Especially in the early months of 2021, it will be vital for local governments to limit unnecessary contact between individuals, and enforce such procedures reliably and consistently. Otherwise, even maximally implemented case detection and vaccination strategies will not be able to protect a significant proportion of the population from infection.

The second category represents the worst-case scenario: *high cost, low impact* strategies. These indicate attempts by governing entities to invest resources in public health interventions, which ultimately still do not effectively control outbreaks. This therefore presents a severe misuse of resources without achieving desired outcomes. Note here that these intervention combinations primarily exclude efficient detection of symptomatic cases. This means that without efficient detection of symptomatic cases—even at full implementation of precautionary quarantine measures, vaccinations, and overall high costs to the economy at large-few infections will be averted.

The final category represents the most favorable category of interventions. Here, *medium cost, high impact* strategies pertain to scenarios involving some investment of resources, directed towards the most efficient policy levers. This results in an effective minimization of infections, thereby constituting well-targeted policy decisions that achieve the objective of controlling the pandemic. Now we see that *both* precautionary measures and efficient symptomatic case detection are vital to achieving this set of outcomes. Even if these interventions do introduce higher costs, they can effectively quell outbreak trajectories early on. Moreover, in these setups, their implementation is even given an allowance for relaxation over time if executed effectively and consistently in the early months. This therefore reduces costs from a broader perspective, as overall fewer infections arise nationally, and fewer resources are demanded to address them.

## Discussion

In this study, we studied optimal strategies for controlling the spread of COVID-19 in the Philippines. We considered existing policy interventions as well as the introduction of vaccination rollouts to quell outbreak trajectories. Dramatic differences were detected in simulated infections depending on which controls were prioritized. In particular, we observed the importance of early and effective implementation of precautionary measures like community quarantines, coupled with efficient detection of symptomatic cases. Furthermore, we found that even if vaccinations alone do not constitute an efficient response to the pandemic, expanding vaccine supply relaxes the need for these more resource-intensive interventions. Meanwhile, although less than ideal, delays in vaccine administration may also be compensated through the remaining policy levers.

These insights bear particular consequences for policies in developing countries like the Philippines [1, 2]. Here, we highlight three key takeaways. First, more than a year into the pandemic, it remains crucial to sustain efficient case detection, isolation, and treatment strategies, particularly for symptomatic cases. In the Philippines, where long-term states of community lockdown have prevailed as the government’s response to short-term fluctuations in COVID-19 cases [39], our findings suggest that an optimal, cost-effective strategy would actually entail relaxations to such measures—but only under the condition that symptomatic case detection is properly implemented. Hence, improving the capacity of the local health system to identify, process, and manage these cases efficiently should be a top priority beyond cyclically adjusting quarantine levels [54].

Second, policymakers need to consider how to expand not just vaccine supply, but also the capacity to administer them. This includes both logistical concerns regarding the strategic use of facilities to vaccinate individuals, inform the public regarding vaccine availability and eligibility, as well as reducing vaccine hesitancy through culturally sensitive health promotion programs that strengthen public trust [3, 55, 56]. Only when such health communication objectives are accomplished and collective behavioral change is initiated can the vaccination strategies posited in this work be made feasible. Otherwise, even procuring sufficient supplies of vaccines will not achieve its intended effects to stop local outbreaks. Initial efforts along these lines are underway by the Philippine National Vaccine Operations Center, for which our model results strongly reaffirm the urgency.

Third, timeliness and consistency must be emphasized in adopting policy measures [4, 5]. Across all favorable scenarios simulated, high levels of key interventions were needed in the early months of 2021, with relaxations projected only mid-2021 or in 2022. Systems for detecting existing cases, while preventing new ones, are needed for vaccinations to meaningfully impact outbreak trajectories and reduce overall costs—especially as these systems need to be robustly sustained in the event of potential delays in acquiring sufficient vaccines for the entire population. This ensures that even if developing countries like the Philippines do not hold sway over the global supply chain of vaccines, the pandemic may still be kept under control through means over which local policymakers do wield authority.

It is worth noting that the conclusions of this work rest on several assumptions. These assumptions constrain the interpretation of our findings but likewise point to promising avenues for future work [6, 7]. A number of limitations pertain to the realism of our model. For instance, we assume a total population that is unaffected by immigration and emigration flows. This can be a source of confounding given that, despite additional precautions in travel protocols, relevant susceptible, exposed, and infectious populations, in reality, include individuals who may leave or arrive within national borders. Furthermore, ordinary differential equation models of diseases, such as the one utilized in this paper, assume homogeneous mixing of individuals in the population. That is, our model assumes that a susceptible individual has a uniform chance of being infected by any infectious individual, regardless of their geographic proximity. However, in an archipelago such as the Philippines, this assumption of free-mixing does not necessarily hold due to different patterns of movement within the country and the tendency of the outbreaks to be concentrated within more urban areas. In relation to this heterogeneity, we further hypothesize that a multi-region approach to mitigation such as in [57] will further lower the total cost of the optimal control strategies. Hence, though we do not include such migratory flows and heterogeneity in our analysis, extensions may valuably consider these factors as well.

We additionally assume that vaccines work by transitioning individuals to a removed compartment. However, it is not the case that all vaccines guarantee 100% immunity for all individuals. Additionally, for parsimony, our model does not incorporate a number of wide-ranging issues which remain pressing to address, yet are beyond the scope of this work. These include: prioritized vaccinations of various segments of the population, lesser-known dynamics of reinfection with COVID-19, the variability in the economic cost of the controls, the impact of emerging variants of the pathogen, documented distinctions between being protected from symptoms while being able to transmit the virus to others, or the practical circumstances of administering vaccines requiring multiple doses [58, 59]. With regard to this latter limitation, we specifically do not model potential logistical impediments in vaccine scheduling or temporary states of partial protection resulting from initial doses [60]. Such considerations therefore place caveats on the implications of our results, further highlighting the need for robust investment in these strategies when translating them into real-world policies. These factors may likewise be modelled with greater precision in succeeding work as growing knowledge continues to accumulate in line with close monitoring by the scientific community [61].

## Conclusions

This study applies optimal control theory to an epidemiological model to calculate the optimal efforts required for precautionary measures, asymptomatic case detection, symptomatic case detection, and vaccination to mitigate the impact of the COVID-19 pandemic. Using parameter values suitable to the Philippines, we show that precautionary measures and symptomatic case detection are essential interventions to minimize infections at an efficient cost. Furthermore, relaxation of measures is feasible after an early and maximal implementation of all controls. Our results also highlight that increasing vaccination capacity and timely acquisition of vaccines are key to reducing the total implementation cost, leading to earlier relaxation of the other non-pharmaceutical interventions in the optimal strategy. This work provides a quantitative reference for drafting policies designed to control the pandemic in the most efficient manner.

## Supplementary Information

**Additional file 1: Appendix S1.** The Appendix is included as a separate file as part of this manuscript submission and contains supplementary information on: (1) Optimality conditions, (2) Control profiles for (ablated) single, dual, and triple control scenarios.

## Data Availability

The data analysed for this study are available in the Department of Health COVID-19 tracker https://www.doh.gov.ph/covid19tracker.

## Abbreviation

COVID-19: Coronavirus disease 2019
