# Supplementary material for: Impact of vaccine supplies and delays on optimal control of the COVID-19 pandemic: mapping interventions for the Philippines
Source: Infect Dis Poverty. 2021 Aug 9;10:107. doi: 10.1186/s40249-021-00886-5 (PMC8352160; doi:10.1186/s40249-021-00886-5)
Supplement: Supplementary file 1 — Additional file 1: Appendix S1. The Appendix is included as a separate file as part of this manuscript submission and contains supplementary information on: (1) Optimality conditions, (2) Control profiles for (ablated) single, dual, and triple control scenarios. [file 40249_2021_886_MOESM1_ESM.pdf]

Appendix:

Supplementary Material for "*Impact of vaccine supplies and delays on optimal control of the COVID-19 pandemic: Mapping interventions for the Philippines*"

Estadilla et al.

Optimality Conditions

The following conditions were used to solve for the optimal control problem in this paper.

Let $\vec{u}^* = (u_1^*, u_2^*, u_3^*, u_4^*)$ be an optimal control of the system given in this paper.

Let f be the integrand of the objective functional and let $X = (S, E, I_a, I_s, C, R)$. By Pontryagin's minimum principle, there exist piecewise differentiable functions $\lambda_j, j = 1, ..., 6$, such that

$$H = f + \lambda_1 \frac{dS}{dt} + \lambda_2 \frac{dE}{dt} + \lambda_3 \frac{dI_a}{dt} + \lambda_4 \frac{dI_s}{dt} + \lambda_5 \frac{dC}{dt} + \lambda_6 \frac{dR}{dt},$$

where

$$\frac{d\lambda_j}{dt} = -\frac{dH}{dX_j},$$

with the transversality condition

$$\lambda_j(t_f) = 0 \text{ for } j = 1, ..., 6,$$

and optimality conditions

$$\begin{aligned}
u_1^* &= \max\left\{u_1^{\min}, \min\left(u_1^{\max}, \frac{S\beta_0(\lambda_2 - \lambda_1)(\psi I_a + I_s)}{2Nw_1}\right)\right\},\\
u_2^* &= \max\left\{u_2^{\min}, \min\left(u_2^{\max}, \frac{(\lambda_3 - \lambda_5)I_a}{2w_2}\right)\right\},\\
u_3^* &= \max\left\{u_3^{\min}, \min\left(u_3^{\max}, \frac{(\lambda_4 - \lambda_5)I_s}{2w_3}\right)\right\},\\
u_4^* &= \max\left\{u_4^{\min}, \min\left(u_4^{\max}, \frac{(\lambda_1 - \lambda_6)\sigma S}{2w_4}\right)\right\}.
\end{aligned}$$

Control Profiles for Single, Dual, Triple Control scenarios

Single control scenarios We implemented optimal control scenarios in which only a single control is available to determine the optimal control profile and the resulting number of infections (Figure 1).

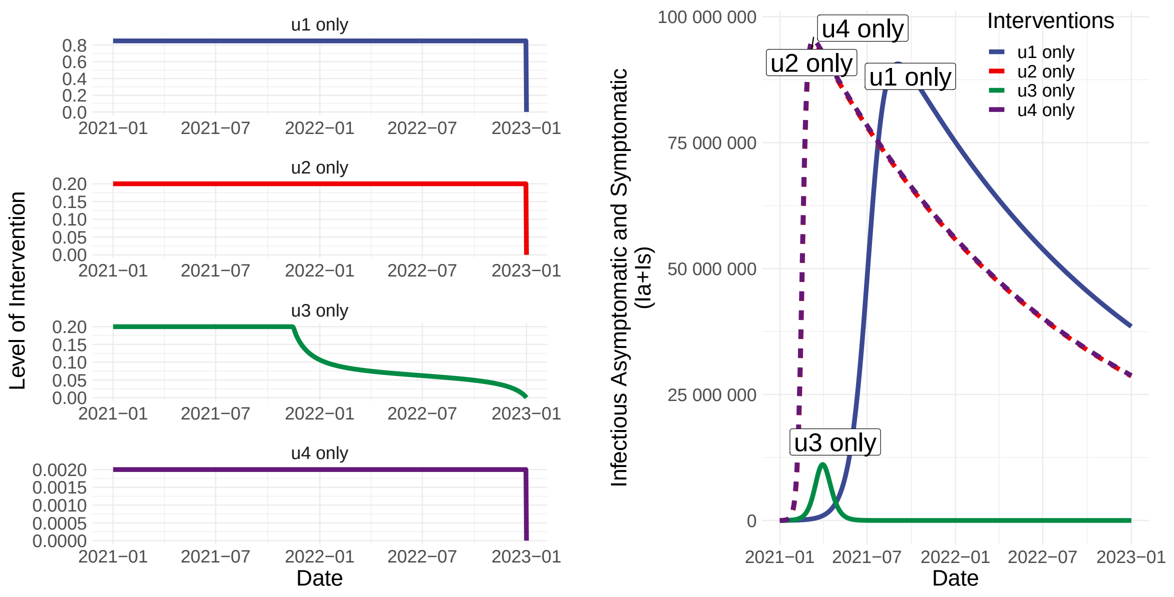

Figure 1: Optimal single control strategies for the COVID-19 epidemic.

For strategies solely relying on just precaution, asymptomatic detection or vaccinations, we observe that all need maximum implementation. They also feature similar patterns in resulting infections, with an extremely steep increase in total infected individuals in the first quarter of 2021, then a gradual decrease throughout the remainder of the two-year period. These curves illustrate that relying only on one of these two strategies is ineffective for both the short and long term, as they cannot keep up with the rate of transmission.

We then observe that a single control strategy of symptomatic detection may be optimized with full implementation until the end of 2021, then gradual easing of testing rates. This results in a significantly reduced projection of total infections relative to the previous single control strategies, with a peak slightly lower than 12 million in the first half of 2021, but overall kept down throughout the rest of the two-year period. This indicates that with just sustained, efficient testing strategies, the pandemic may be controlled. However, we note that around the peak level of infection, several million symptomatic cases would have to be detected each day, pointing to an even larger number of tests. Thus, although theoretically possible, a single control strategy may still impose strain on the national health system.

Dual control scenarios We also consider the implementation of dual control strategies and compare each scenario by the resulting optimal control profiles (Figure 2) and the projected number of infected

individuals (Figure 3). The key observation from this analysis is that the dual controls which involve precautions and symptomatic detection result in exponentially smaller numbers of infections, with the best case observed with both controls together. In contrast, when implementing only asymptomatic detection and vaccinations, maximum levels of both controls are required, yet an extremely high peak of 90 million cases is observed as early as the first quarter of 2021.

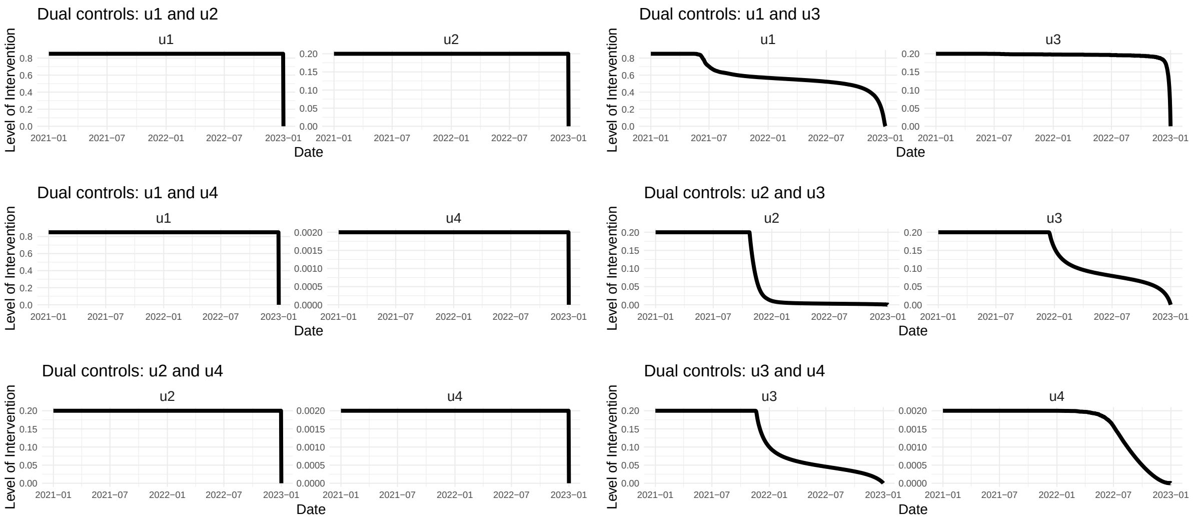

Figure 2: Optimal dual control strategies for the COVID-19 epidemic.

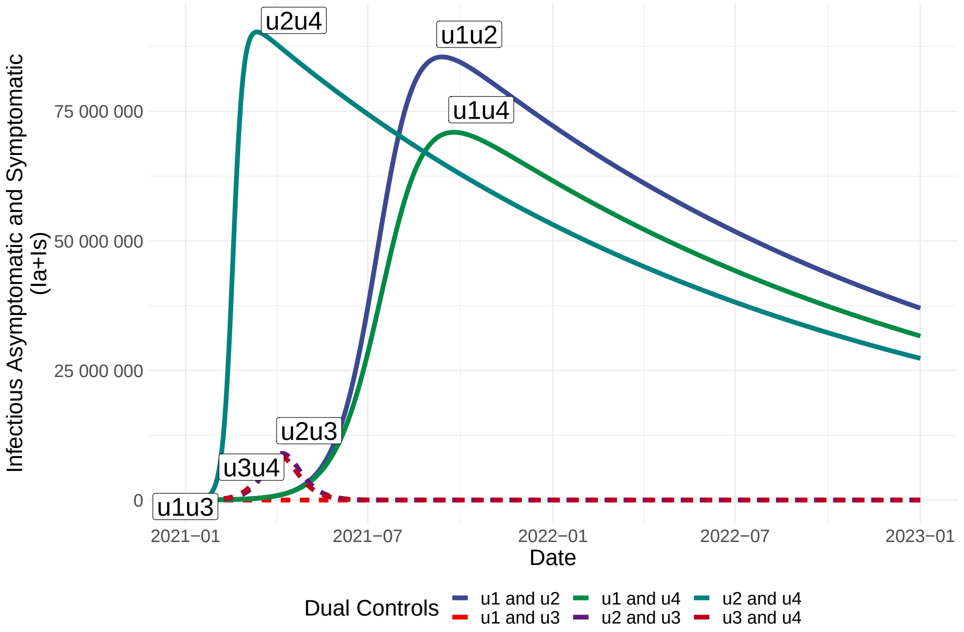

Figure 3: Total infectious individuals ($I_a + I_s$) per optimal dual control strategy for the COVID-19 epidemic.

Meanwhile, relying only on precaution and asymptomatic detection, or only on precaution and vaccination likewise require maximum levels of each control. Although they result in lower peaks than other

scenarios, the requirement of maximum maintenance of lockdown measures throughout the two-year period nonetheless presents a major hurdle for government implementation as well as severe economic strain.

By contrast, the best dual control strategy entails a sustained maximum capacity symptomatic detection efficiency combined with gradually loosened precaution measures. This strategy results in the least number of infected individuals, with a peak lower by orders of magnitude than all others. The remaining two dual control strategies feature full-blown symptomatic detection strategies for the entire 2021, then gradual reductions in 2022. These are combined with either full capacity asymptomatic detection for the duration of 2021, which are subsequently stopped in 2022; or a full capacity vaccination effort for the majority of the entire two-year period. Both experience moderate peaks by the first quarter of 2021, comparable to more lockdown-intensive procedures without the same severe restrictions on economic activity.

Triple control scenarios Finally we examine the optimal control profiles when only three controls are available (Figure 4) and the resulting number of total infections 5. Alternatively, these may be conceived as leave-one-out strategies, whereby scenarios are compared based on which of the four controls is omitted. Aligning with the analysis in the previous section, we observe that leaving out symptomatic detection results in a peak of nearly 70 million infections in the second half of 2021. While we note this is lower than the other extreme peaks detected in single and dual control scenarios, we see consistently that neglecting efficient symptomatic detection—even at full implementation of all the remaining controls—results in worse outcomes than other scenarios.

If precautions are left out of the intervention strategy, we notice the highest peak at about 7 million infected individuals in the first half of 2021. But cases remain low for the next two years following this peak period. To achieve this, both asymptomatic and symptomatic detection have to be at full capacity for 2021, but may be eased by 2022. Vaccination efforts, however, have to be sustained at their maximum levels until near the end of the two-year period. While this strategy thus eases economic restrictions, it nonetheless entails the second-highest health costs among the triple control scenarios.

The remaining triple control scenarios illustrate the combined effectiveness of precautions and efficient symptomatic detection. For both scenarios, the peaks are not visually discernible in the graphs because they are both orders of magnitude below the other scenarios. If precautions and symptomatic detection are combined with asymptomatic detection, then symptomatic detection has to be sustained at maximum capacity for the entire two year period. Precautions may then start to ease as early as the first quarter of 2021, then kept at a moderate level for the remainder of the two-year period. Asymptomatic detection, on the other hand, only needs to be at maximum capacity for the first quarter, then held at minimal levels for the remainder of the two-year period.

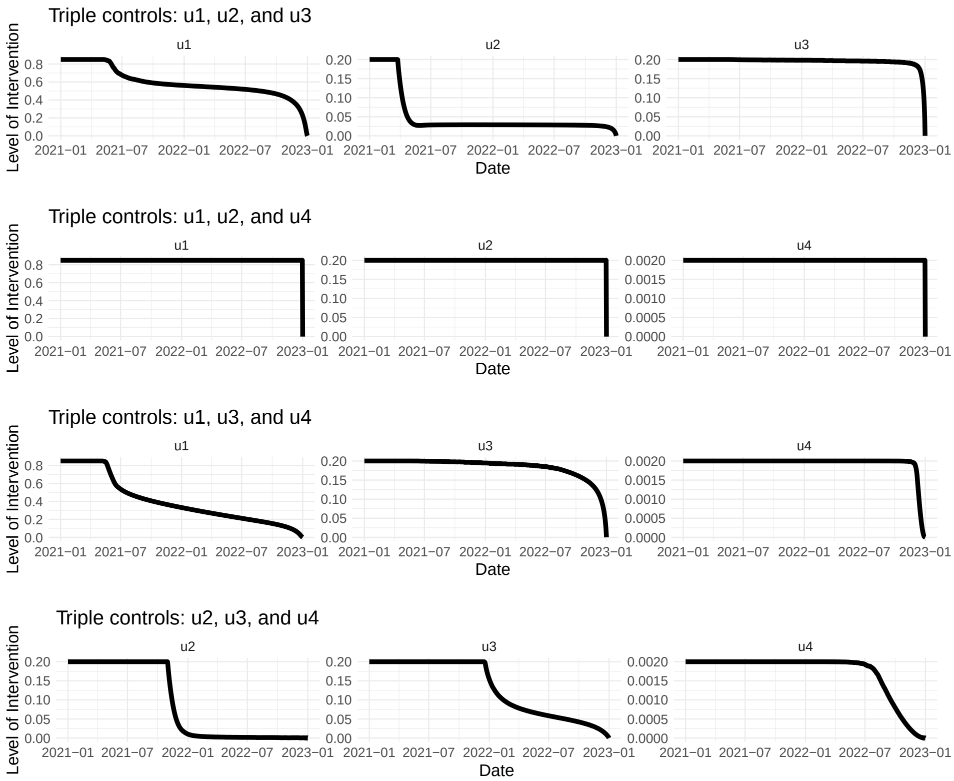

Figure 4: Optimal triple control strategies for the COVID-19 epidemic.

Meanwhile, if vaccinations accompany both precautions and symptomatic detection, then both symptomatic detection and vaccinations have to be kept at maximum capacity for the entire two-year period. But as before, precautions only need to be at maximum for the first quarter of 2021, then may be kept at a moderate level up to the end of 2022.

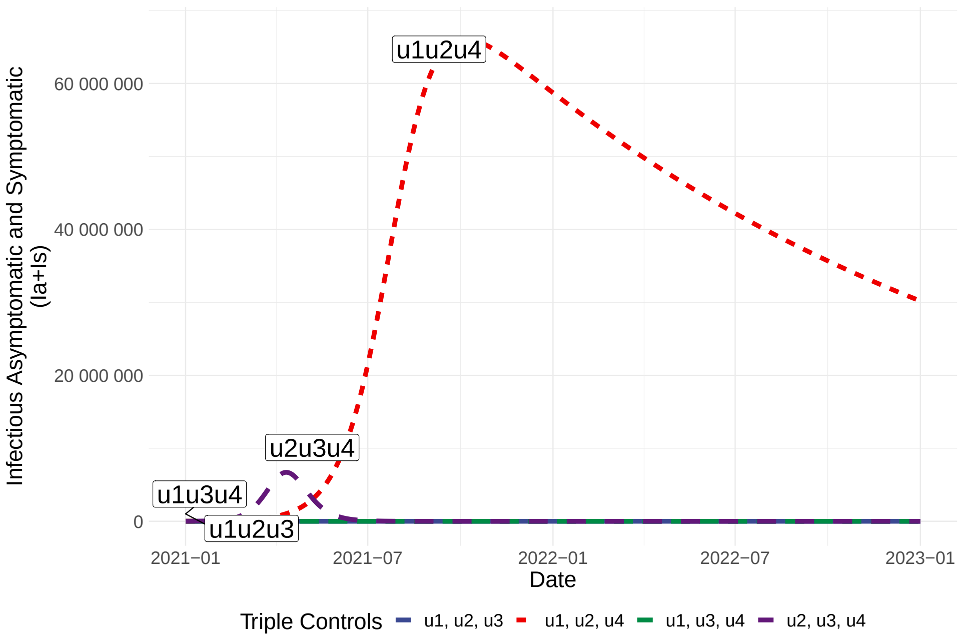

Figure 5: Total infectious individuals ($I_a + I_s$) per optimal triple control strategy for the COVID-19 epidemic.
